# Supplementary material for: Structures of the human peroxisomal fatty acid transporter ABCD1 in a lipid environment
Source: Commun Biol. 2022 Jan 10;5:7. doi: 10.1038/s42003-021-02970-w (PMC8748874; doi:10.1038/s42003-021-02970-w)
Supplement: Supplementary file 2 — Supplementary Information [file 42003_2021_2970_MOESM2_ESM.pdf]

Title **Supplementary Information:**

Structures of the human peroxisomal fatty acid transporter ABCD1 in a lipid environment

Authors

Le Thi My Le, James Robert Thompson, Phuoc Xuan Dang, Janarjan Bhandari & Amer Alam*

Supplementary Figure 1.

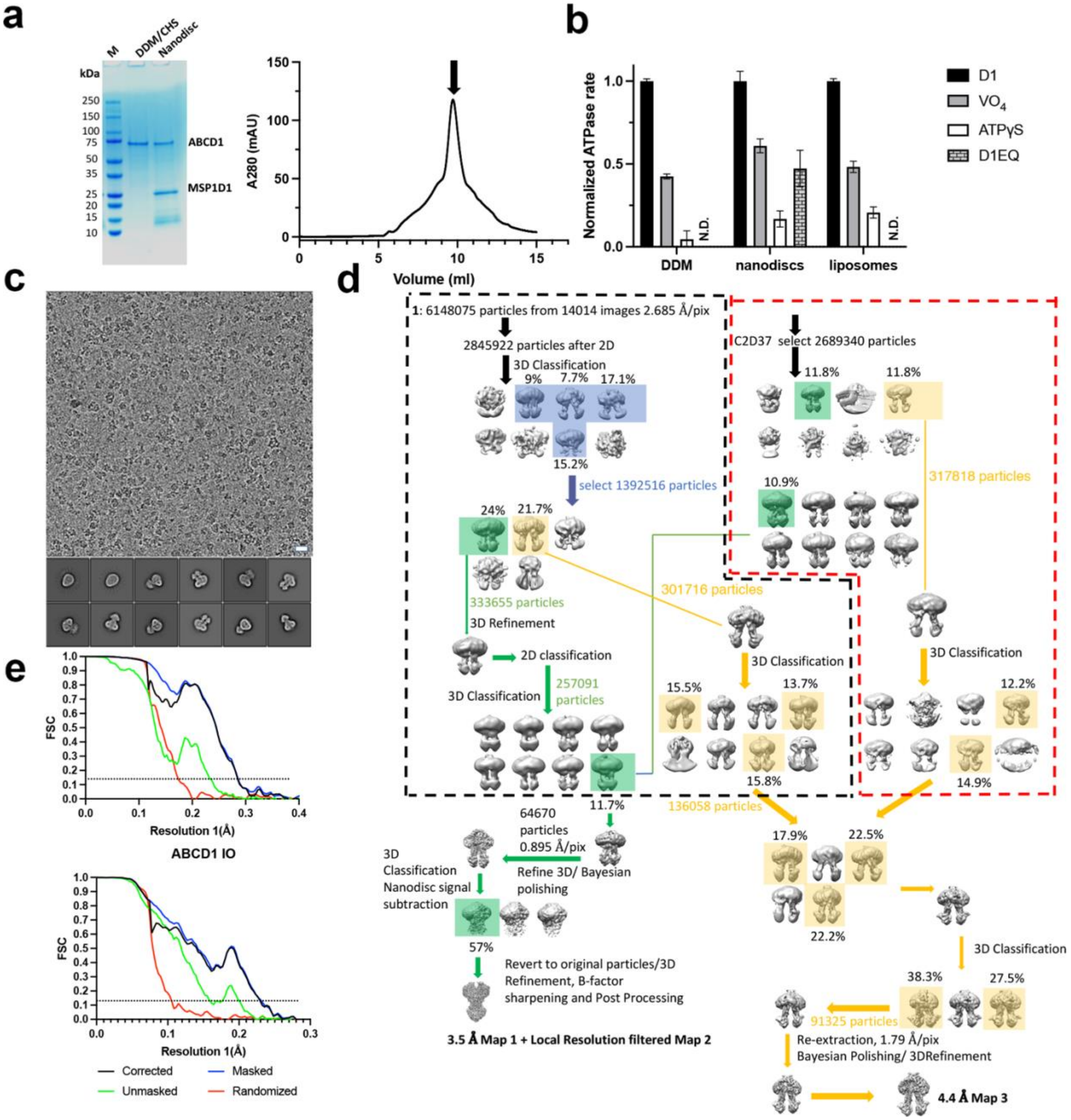

Supplementary Figure 1: Functional reconstitution and structure determination of human ABCD1. (**a**) 4-15% Coomassie stained gradient gel and SEC profile for nanodisc reconstituted ABCD1 (**b**) ATPase activity of wild type ABCD1 (D1) and $ABCD1_{EQ}$ mutant (D1EQ) in the presence of sodium orthovanadate (VO4) and ATP*γ*S normalized to activity of wildtype ABCD1 alone. N=3, error bars represent S.D. N.D=not determined. (**c**) Representative micrograph of nanodisc reconstituted ABCD1 at ~2.5 µM defocus along with representative 2D classes. Scale bar equals 20 nm. (**d**) Data processing flowchart. Classes leading to OO and IO structures are highlighted green and yellow, respectively. Dashed lines represent individual datasets before classes were combined for further processing. (**e**) Fourier Shell correlation (0.143 cutoff dashed line) curves for ABCD1 IO and OO maps.

Supplementary Figure 2.

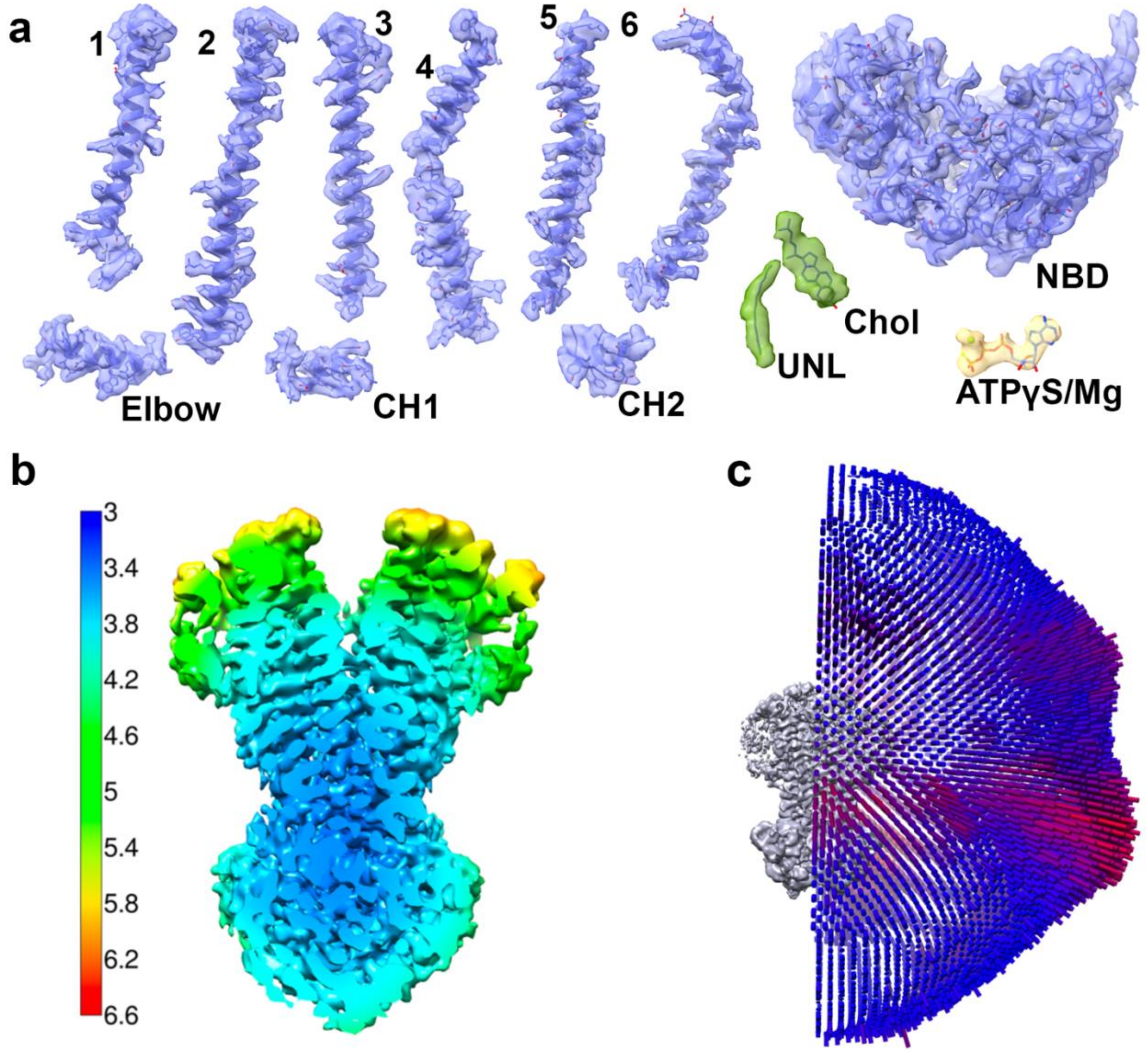

Supplementary Figure 2: (**a**) Map and model for individual TMs, Elbow Helix, Coupling helices (CHs), and NBD of the ABCD1 OO structure, along with modeled acyl chain (UNL for unknown ligand), cholesterol (Chol), and ATPγS/Mg. All Maps are displayed at a contour level of 0.025 except for ATPγS/Mg (0.075 contour). (**b**) Central slice through local resolution filtered EM map of nanodisc reconstituted human ABCD1 OO with resolution color key at left. (**c**) Angular distribution plot with refined map shown for reference.

Supplementary Figure 3.

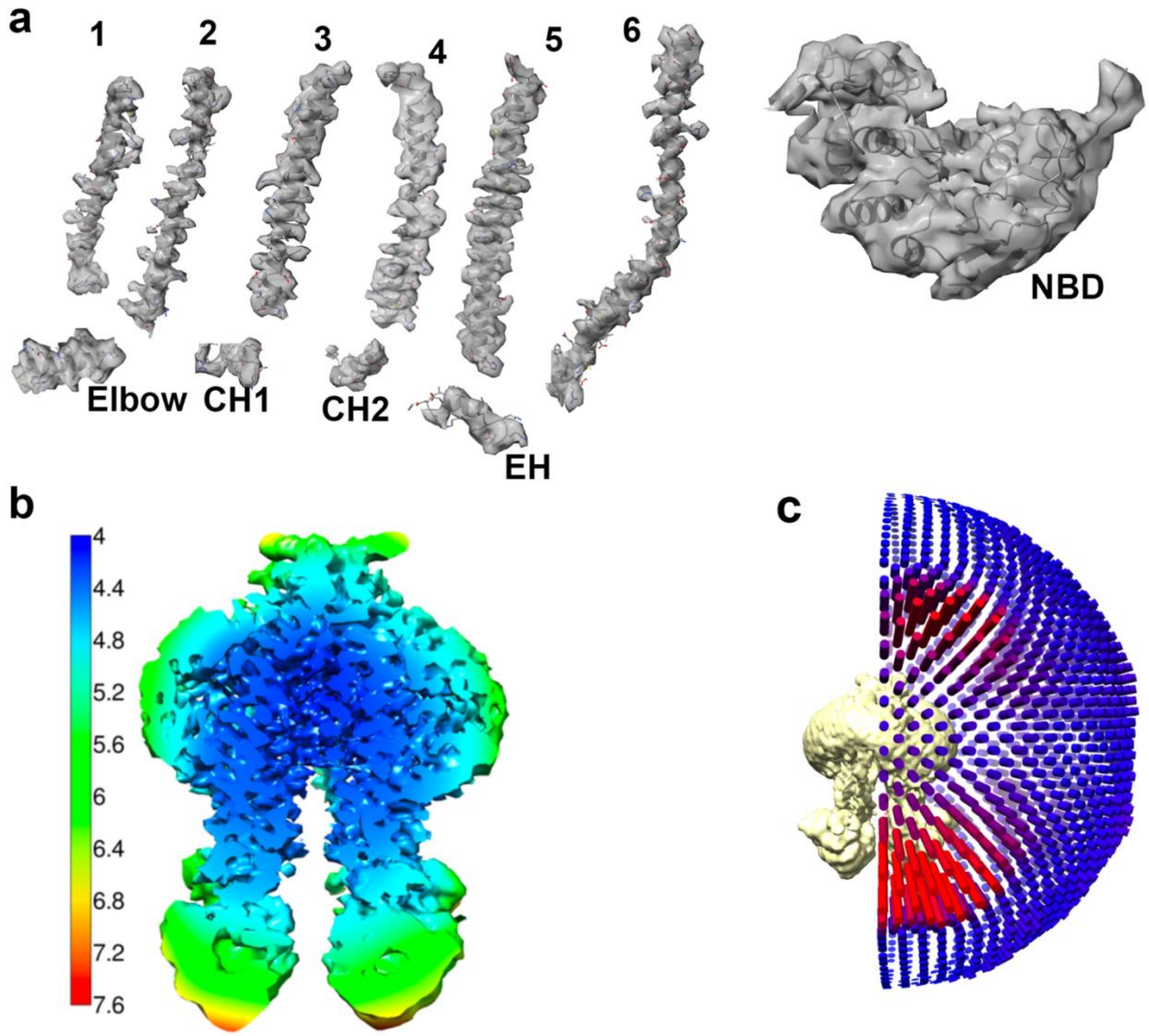

Supplementary Figure 3: (**a**) Map (0.05 contour) and model for individual TM, Elbow, CH, EH helices and NBD of the ABCD1 IO structure. (**b**) Central slice through local resolution filtered EM map of nanodisc reconstituted human ABCD1 IO with resolution color key at left. (**c**) Angular distribution plot with refined map shown for reference.

Supplementary Figure 4.

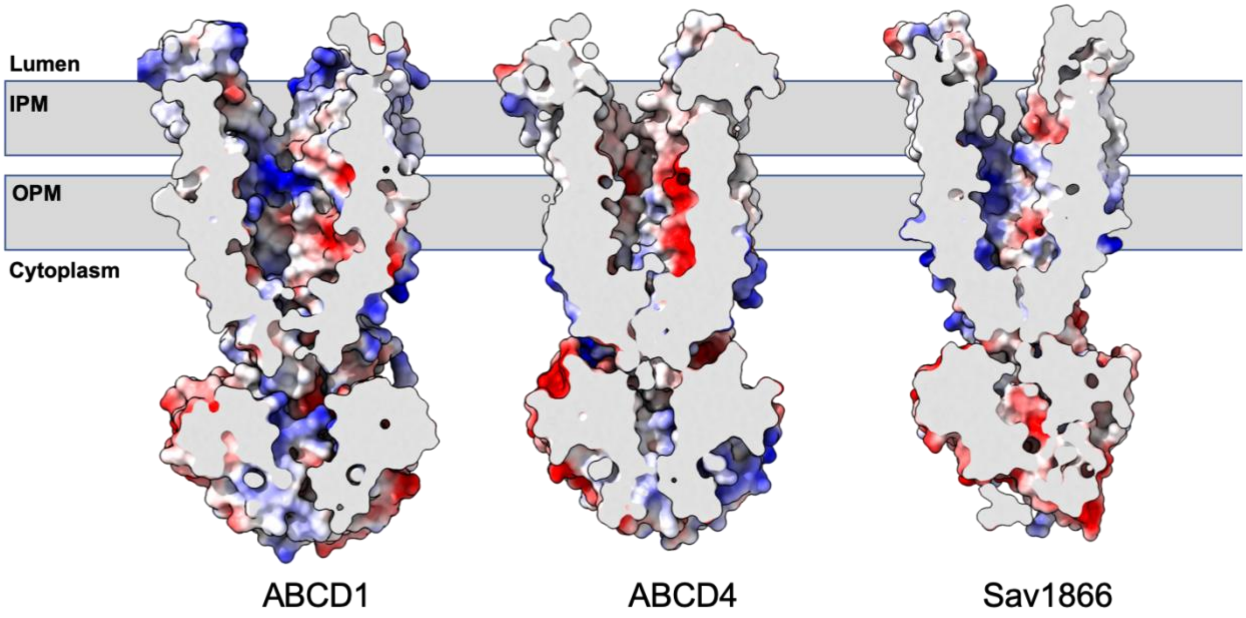

Supplementary Figure 4: Comparison of ABCD1 transmembrane cavity to that of ABCD4 and Sav1866, showing electrostatic surface. Grey bar represents approximate position of peroxisomal membrane. IPM= Inner peroxisomal membrane, OPM=outer peroxisomal membrane.

Supplementary Figure 5.

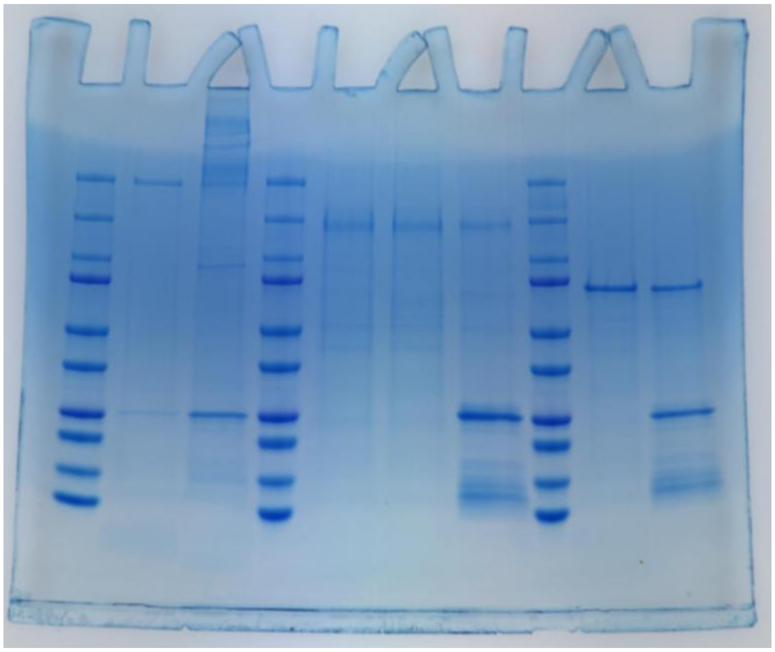

Supplementary Figure 5: The entire 4-15% Coomassie stained gradient gel that corresponds to the cropped image shown in Supplementary Figure 1a above. Only gel lanes 8-10 are relevant to the research presented in this manuscript.

Supplementary Table 1.

Dataset	Nanodisc reconstituted ABCD1	
Magnification	96k	
Pixel Size (Å)	0.895	
Total Dose (e/Å^2)	60	
Defocus Range (um)	-0.8 to 2.6	
Maps	Human ABCD1 OO	Human ABCD1 IO
EMDB ID	EMD-24656	EMD-24657
# Particles in final Class	37237	91325
Resolution (Å) (0.143 threshold)	3.5	4.4
Sharpening B factor	-65	-50
**Refined Coordinates**	Human ABCD1 OO	Human ABCD1 IO
PDB ID	7RR9	7RRA
# Residues/Non-hydrogen Atoms<br>Ligands	1120/9096<br>8	1204/9610
R.M.S deviations<br>Bond Length (Å)<br>Bond Angles (°)	 0.003<br>0.591	 0.003<br>0.593
MolProbity Statistics<br>MolProbity Score<br>Clashscore<br>Poor rotamers (%)<br>Z-Score<br>Whole<br>Helix<br>Sheet<br>Loop	 1.67<br>7.16<br>0.00<br><br>1.00<br>1.41<br>1.08<br>-1.53	 1.67<br>9.31<br>0.00<br><br>1.98<br>2.17<br>1.98<br>-1.51
EM Ringer Score	1.6	0.635
Ramachandran statistics<br>Favored (%)<br>Allowed (%)<br>Outliers (%)	 96.03<br>3.97<br>0.00	 96.99<br>3.01<br>0.00

Supplementary Table 1: Data processing and refinement statistics
ABCD1 OO refers to outward-open conformation Cryo-EM dataset for human ABC transporter D1, while IO refers to data for inward-open conformation. Identification numbers for EMDB and PDB refer to EM Data Bank and Protein Data Bank, respectively.

Supplementary Figure 6.

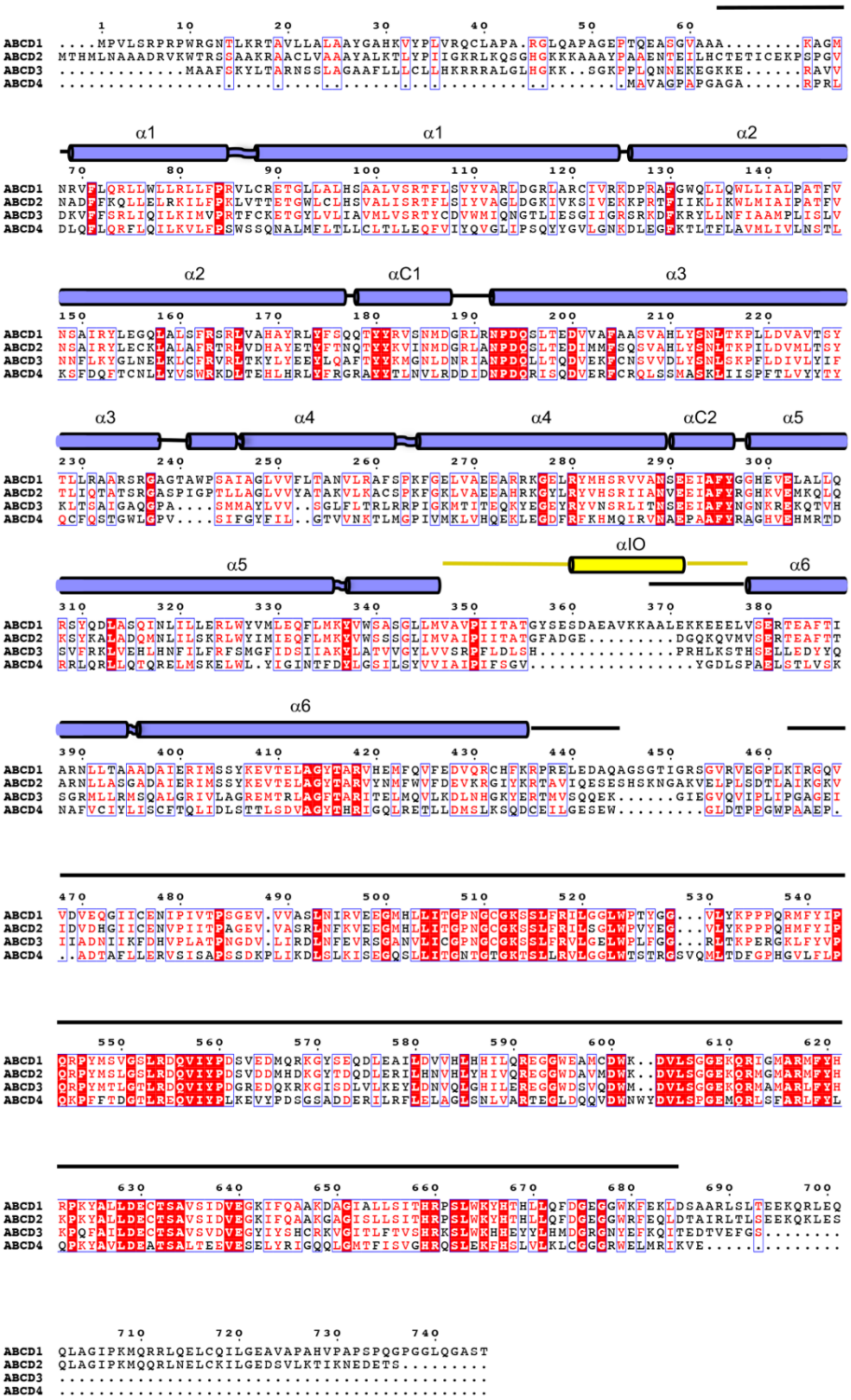

Supplementary Figure 6: Sequence alignment of human ABCD family transporters with secondary structure assignment based on ABCD1 structures presented in this study shown.
