# Supplementary material for: Structures of the human peroxisomal fatty acid transporter ABCD1 in a lipid environment
Source: Commun Biol. 2022 Jan 10;5:7. doi: 10.1038/s42003-021-02970-w (PMC8748874; doi:10.1038/s42003-021-02970-w)
Supplement: Supplementary file 3 — Description of Additional Supplementary Files [file 42003_2021_2970_MOESM3_ESM.pdf]

Description of Additional Supplementary Files

File name: Supplementary Data 1
Description: ABCD1 ATPase Activity Data used in making Figure graphs.
